# Torsion of Fatty Appendage of Falciform Ligament: Acute Abdomen in a Child

**DOI:** 10.1155/2015/293491

**Published:** 2015-11-18

**Authors:** Caroline Maccallum, Sarah Eaton, Daniel Chubb, Stephen Franzi

**Affiliations:** ^1^Department of General Surgery, Royal Melbourne Hospital, Melbourne, VIC 3050, Australia; ^2^Department of General Surgery, Northeast Health Wangaratta, Wangaratta, VIC 3677, Australia

## Abstract

Torsion of the fatty appendage of the falciform ligament is an extremely rare condition that leads to severe abdominal pain and raised inflammatory markers. It can be recognised on ultrasound or CT scan. The pathophysiology is the same as that involved in the more common torsion and/or infarction of the greater omentum or epiploic appendages. The condition is best managed conservatively with anti-inflammatory analgesia, and the early recognition of this type of torsion may prevent unnecessary operative intervention to look for a source of abdominal pain. There have been five reported adult cases of a torted fatty appendage of the falciform ligament identified on ultrasound and CT scan, but no paediatric cases. We report a case of torsion of the fatty appendage of the falciform ligament in a ten-year-old boy and describe its imaging characteristics on CT scan.

## 1. Introduction

The falciform ligament is a double fold of peritoneum that marks the anatomical division between the right and left lobes of the liver. Pathologic conditions of the falciform ligament are extremely rare; one particularly rare condition is the torsion of a fatty appendage of the falciform ligament leading to fat infarction [1, 2]. This type of torsion and/or infarction occurs more commonly in the greater omentum or epiploic appendages [3]. The condition causes abdominal pain and associated raised inflammatory markers, and it can be identified on ultrasound and CT scan. To our knowledge, there have only been five reported adult cases of a torted fatty appendage of the falciform ligament identified on ultrasound or CT scan, but there are no paediatric cases.

In this paper, we report on a paediatric case of a torted fatty appendage of the falciform ligament as seen on CT and discuss the best management options for this type of patient.

## 2. Case Presentation

A 10-year-old boy presented with five days of right sided abdominal pain associated with vomiting, diarrhoea, and anorexia. The patient's medical history included craniosynostosis, attention deficit hyperactivity disorder, asthma, and migraines.

On examination, the patient had tenderness and voluntary guarding in the right upper quadrant, and McBurney's sign was negative. The patient was afebrile and was not jaundiced. His admission blood tests showed a high white cell count (21.4 × 10^9^/L), with no left shift. Liver function tests, C-reactive protein, and amylase were unremarkable.

The abdominal ultrasound showed no evidence of cholelithiasis or cholecystitis, and the diameters of the intrahepatic and extrahepatic bile ducts were within normal range. The appendix was not visualised and no free fluid was present. At the time of reporting, there were no abnormalities identified in the area of the liver or the falciform ligament.

**Figure 1:**
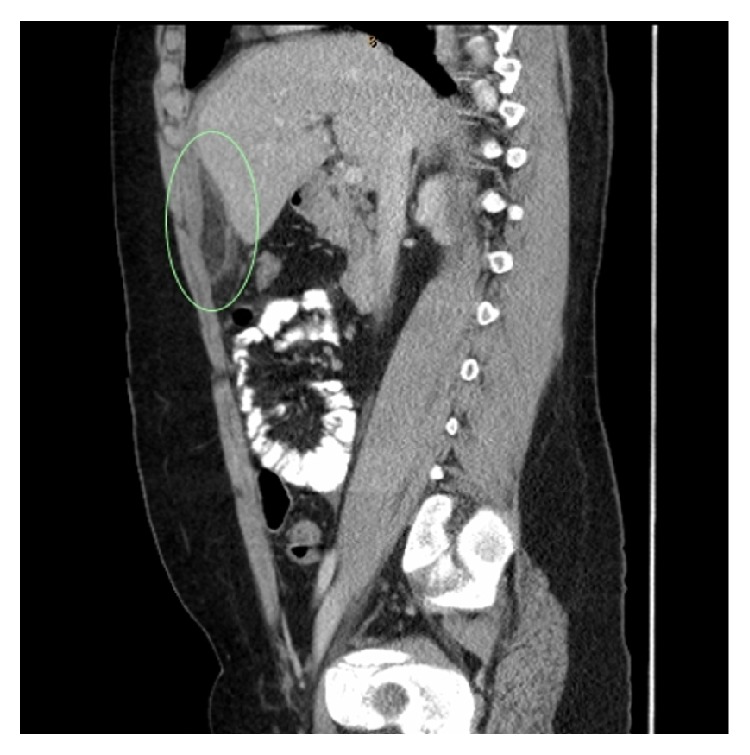
CT abdomen and pelvis-sagittal view. Torsion of lipomatous appendage of falciform ligament circled.

**Figure 2:**
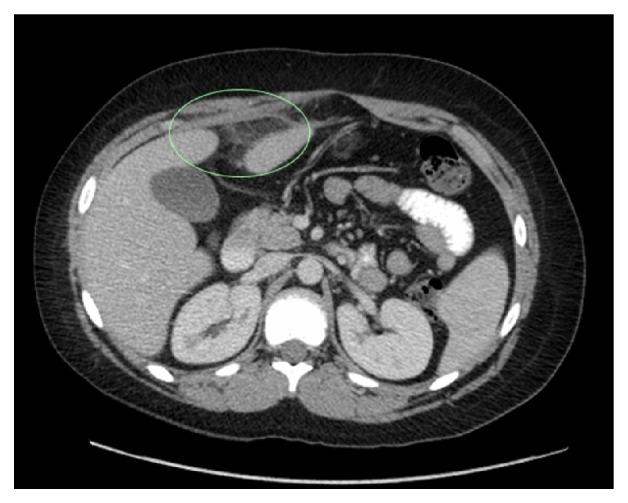
CT abdomen and pelvis, coronal view. Torsion of lipomatous appendage of falciform ligament circled.

The patient was admitted and observed for possible early appendicitis. The inflammatory markers returned to normal, but the abdominal pain persisted. Given the atypical presentation, computed tomography (CT) of the abdomen and pelvis with intravenous and oral contrast was performed. The CT showed hazy increased density of fat and inflammatory changes centred around a focal area of fat, which was anterior and inferior to the left lobe of the liver and adjacent to the falciform ligament. These features suggested incarcerated fat with inflammatory change. These radiological signs are similar to those recognised for epiploic appendagitis and omental infarction, which appear as areas of focal fat infarction and local inflammatory changes, elsewhere in the abdomen. In view of the site and the relevant literature, this CT was in keeping with torsion of a fatty appendage of the falciform ligament (Figures 1 and 2).

Observation of the patient continued, with oral analgesia used to control pain. The patient did not have an exploratory laparotomy or laparoscopy. The pain resolved after four days, and the patient was then discharged. On review two months later, the pain had mostly resolved.

## 3. Discussion

The falciform ligament is a double fold of peritoneum that anatomically divides the liver into the right and left lobes. It extends from the superior edge of the liver to the inferior border of the diaphragm. It contains the ligamentum teres, paraumbilical veins, and variable amount of extraperitoneal fat. The falciform ligament receives its arterial supply from a vessel coming off the left inferior phrenic artery and the middle segmental artery of the liver [4].

It is extremely rare to see pathologic conditions of the falciform ligament. Recognised conditions include ligament cysts, tumours, abnormal vascularisation due to portal hypertension, iatrogenic internal hernia through the ligament, and gangrene related to acute necrotising pancreatitis, along with torsion of a fatty appendage of the falciform ligament as described in this case [1, 2].

The term intra-abdominal focal fat infarction (IFFI) has been used to describe focal lipomatous tissue necrosis in various anatomical locations [4]. IFFI are most often due to torsion and/or infarction of the greater omentum or epiploic appendages but have also been reported to involve the lesser omentum and the lipomatous appendage of the hepatic falciform ligament.

Both ultrasound and CT can be used to visualise a torted fatty appendage of the falciform ligament. It is not possible to use plain film radiography to diagnose this condition, as the falciform ligament is only evident on abdominal plain films in the setting of pneumoperitoneum [5, 6]. In that situation, the “falciform ligament sign” is produced, which consists of gas outlining the falciform ligament. On ultrasound, a torted fatty appendage of the falciform ligament appears as a hyperechoic, noncompressible, slightly heterogenous mass in the area of maximal abdominal tenderness [7]. Further, on real-time sonography, the lesion does not move with underlying intraperitoneal structures while breathing, which indicates its extraperitoneal position [8]. On CT, a torted fatty appendage of the falciform ligament appears as an area with increased fat density, associated with surrounding inflammatory changes in the adjacent fat planes [7]. To our knowledge, there has been no previous description of the appearance of a twisted infarcted fatty appendage of the falciform ligament on MRI. However, MRI would be a valid alternative form of imaging to diagnose this condition, because it would distinguish adipose tissue from oedema or bleeding, it avoids radiation exposure particularly in the case of a paediatric patient, and it avoids contrast medium administration and its associated nephrotoxicity.

According to our literature search, there have only been five reported adult cases of a torted fatty appendage of the falciform ligament identified on ultrasound and/or CT scan, with our case being the first paediatric case reported [1, 2, 7–9] (there were three earlier case reports of this pathology, but, as they were reported before 1977, there were no ultrasound or CT images) [10–12]. Each adult case presented with upper abdominal pain, with varying combinations of right upper quadrant, epigastric region, and left upper quadrant pain, and there were three cases with a mild increase in CRP or leucocytosis.

CT was diagnostic in each case, and the diagnosis was confirmed in 80% of the cases with an exploratory laparotomy. In each paper, there was consistency in the CT appearance of a torted fatty appendage of the falciform ligament. A typical image showed an area of fat density with focal inflammatory changes in the local fat, in the area of the falciform ligament.

In terms of treatment, Coulier conducted a review of IFFI (including cases involving the falciform ligament) and argued that in most cases the patient improves with conservative management and that surgical intervention is not required [3]. This is particularly the case given the high quality of CT scans, which make it possible to identify an IFFI on imaging rather than requiring intraoperative characterisation.

Although rare, our observation of a torted lipomatous appendage of the falciform ligament or IFFI should be considered as a part of a differential diagnosis when children and adults present with atypical right upper quadrant pain. This is particularly important because it may prevent the patient from having unnecessary surgery.
